# Highly glycosylated CD147 promotes hemorrhagic transformation after rt-PA treatment in diabetes: a novel therapeutic target?

**DOI:** 10.1186/s12974-019-1460-1

**Published:** 2019-04-05

**Authors:** Yanan Xie, Yingzhe Wang, Hongyan Ding, Min Guo, Xun Wang, Qiang Dong, Mei Cui

**Affiliations:** 1 Department of Neurology, Huashan Hospital, Fudan University, No.12 Middle Wulumuqi Road, Shanghai, 200040 China; 2 The State Key Laboratory of Medical Neurobiology, Fudan University, Shanghai, China; 3 Department of Neurology, Jing’an District Centre Hospital of Shanghai, Shanghai, China

**Keywords:** Diabetes, Ischemic stroke, Blood-brain barrier, Glycosylation, CD147, Glucagon-like peptide-1 receptor agonist

## Abstract

**Background:**

Diabetes is known to be a main risk factor of post-stroke hemorrhagic transformation following recombinant tissue plasminogen activator (rt-PA) therapy. However, the mechanism through which diabetes exacerbates hemorrhagic transformation is insufficiently understood. We aimed to verify that CD147, the extracellular matrix metalloproteinase (MMP) inducer, played a vital role in the progress.

**Methods:**

We performed middle cerebral artery occlusion on diabetic and non-diabetic rats, with or without rt-PA treatment, and then compared the glycosylation level of CD147, caveolin-1, MMPs activities, and blood-brain barrier (BBB) permeability. In vitro*,* tunicamycin treatment and genetic tools were used to produce non-glycosylated and lowly glycosylated CD147. An endogenous glucagon-like peptide-1 receptor (GLP-1R) agonist was used to downregulate the glycosylation of CD147 in vivo.

**Results:**

Compared with non-diabetic rats, diabetic rats expressed higher levels of highly glycosylated CD147 in endothelium and astrocytes following rt-PA treatment accompanied by higher activity of MMPs and BBB permeability, in the middle cerebral artery occlusion model. Caveolin-1 was also overexpressed and co-localized with CD147 in astrocytes and endothelium in diabetic rats. In vitro, advanced glycation end products increased the expression of highly glycosylated CD147 in astrocytes and endothelial cells. Downregulating the glycosylation of CD147 lowered the activity of MMPs and promoted the expression of tight junction proteins. The expression of caveolin-1 in endothelial cells and astrocytes was not inhibited by tunicamycin, which revealed that caveolin-1 was an upstream of CD147. In vivo, GLP-1R agonist downregulated the glycosylation of CD147 and further reduced the activity of MMPs and protected the BBB in diabetic rats.

**Conclusion:**

CD147 is essential for diabetes-associated rt-PA-induced hemorrhagic transformation, and downregulation of CD147 glycosylation is a promising therapy for neurovascular-unit repair after rt-PA treatment of patients with diabetes.

**Electronic supplementary material:**

The online version of this article (10.1186/s12974-019-1460-1) contains supplementary material, which is available to authorized users.

## Background

Diabetes mellitus (DM) has become a worldwide health challenge as the number of DM patients has reached 415 million and may grow up to 642 million by 2040 [1]. DM substantially increases the risk of first ischemic stroke by two to four times [2]. Acute ischemic stroke is now one of the most severe comorbidities of DM and the leading cause of disability in DM patients. In China, approximately 400,000 diabetic patients suffer cerebral infarction every year which places a heavy financial burden on society [3]. Thrombolysis therapy promotes reperfusion in ischemic stroke patients during the hyperacute period which ultimately improves prognosis. Recombinant tissue plasminogen activator (rt-PA) has been certificated by multiple clinical trials as being a beneficial thrombolytic drug for the treatment of ischemic patients [4–6]; however, its clinical application requires caution due to the severe side effects which have also been reported. Approximately 6% of ischemic stroke patients who have received rt-PA treatment will suffer symptomatic intracranial hemorrhagic transformation (HT), which can subsequently lead to severe disability and even death [7]. Unfortunately, DM/stress hyperglycemia is found to be a main risk factor of rt-PA-induced symptomatic intracranial HT and a worse clinical outcome [8–11]. The aim of this study is to understand how DM/hyperglycemia exacerbates rt-PA-induced HT and investigate potential solutions for the application of rt-PA in DM patients with ischemic stroke.

Several studies have demonstrated that after rt-PA treatment, matrix metalloproteinases (MMPs), predominantly MMP-2 and MMP-9, decompose tight junction proteins and extracellular matrix, causing neurovascular unit damage, blood-brain barrier (BBB) disruption, and subsequently HT [12–14]. DM patients exhibit higher levels of serum MMPs, which may contribute to the exacerbation of HT [12, 15]. CD147, also known as extracellular matrix metalloproteinase inducer (EMMPRIN), is a type-I transmembrane glycoprotein of the immunoglobulin superfamily (IgSF), which functions as known as an MMP inducer and is widely expressed by human tumor cells [16]. The glycosylation of CD147 is critical for its MMP-inducing activity. Highly glycosylated CD147 (HG-CD147), rather than the lowly glycosylated form (LG-CD147), plays a crucial role in MMP induction [17]. Consistently, DM patients exhibit a higher level of highly glycosylated CD147 in the periphery [18]. Although the status of CD147 and MMPs in the central nervous system (CNS) remains undescribed, it is plausible to hypothesize that DM increases the level of HG-CD147 and thus the activity of MMPs in the CNS, causing the exacerbation of HT after rt-PA treatment of ischemic stroke. In this article, we try to verify this hypothesis and further explore the underlying molecular mechanisms.

## Methods

### Animal models and treatment

This study was carried out following the Guide for the National Science Council of the People’s Republic of China. Eight-week-old male Wistar rats with initial body weight of 200–220 g were randomized into two groups: diabetes and non-diabetes. The non-diabetes group was injected with saline, while the diabetes group received a standard intraperitoneal injection of streptozotocin (60 mg/kg; Sigma, St. Louis, MO, USA) to induce diabetes. Seven days after streptozotocin administration, rats with a blood glucose concentration of > 15 mmol/L were retained as successfully fitting the model as previously described [19].

Both groups were subjected to middle cerebral artery occlusion (MCAO) to generate an ischemic stroke model as described previously [20]. In brief, the rat was anesthetized with ketamine/xylazine (65/6 mg/kg) and put onto a heating pad and feedback control system (FHC, Bowdoin, ME, USA) to keep the body temperature at 37 °C. A siliconized filament was inserted into the internal carotid artery and advanced until the origin of the middle cerebral artery to produce a sudden drop of cerebral blood flow (CBF) to below 25% of baseline measured using a laser Doppler monitor (Periflux PF3, Perimed, Stockholm, Sweden). After 60 min, the filament was removed.

Immediately after reperfusion, rt-PA (10 mg/kg) or saline was administered as an intravenous bolus injection of 1 mg/kg followed by a 9 mg/kg infusion for 30 min with a syringe infusion pump (World Precision Instruments). Thus producing four groups: diabetes + saline, diabetes + rt-PA, non-diabetes + saline, and non-diabetes + rt-PA.

To examine the effectiveness of Exendin-4, rats were randomized into four groups: control + saline, control + rt-PA, Ex-4 + saline, Ex-4 + tr-PA (*n* = 6 each group). All animals were subjected to MCAO, and the administration of rt-PA was performed as described above. Exendin-4 (Sigma-Aldrich, USA; 10 mg/kg) was administrated by the caudal vein before rt-PA or saline injection [21].

### Assessment of infarct volume and blood-brain barrier permeability

The infarct volume was measured 72 h after MCAO using 2,3,5-triphenyltetrazolium chloride (TTC, Sigma). Only those rats in which CBF below 25% of baseline was achieved and with a return to > 80% after reperfusion were included. Infarct volume was calculated as described previously [22, 23]. To measure BBB permeability, Evans blue dissolved in saline (*m*/*v*, 2%) was injected into the caudal vein 72 h after MCAO. Three hours following injection, animals were sacrificed by an overdose of anesthetic and immediately were subjected to transcardiac perfusion with saline. Then brain hemispheres were removed and homogenized in 3 mL of N,N-dimethylformamide (Sigma-Aldrich), followed by incubation for 18 h at 55 °C and subsequent centrifugation. The supernatants were subjected to spectrophotometry at 620 nm.

### Quantification of cerebral hemorrhage

The hemoglobin content in brain tissues was quantified by spectrophotometric assay. The hemispheric brain tissue was homogenized with PBS and centrifuged for 30 min (13,000 g). The hemoglobin-containing supernatants were collected. A 20-μL aliquot of supernatant was mixed with 80 μL of Drabkin’s reagent (Sigma Diagnostics), followed by incubation at room temperature for 15 min followed by optical density (OD) measurement at 540 nm. The hemorrhage volume was expressed in equivalent units by comparing OD values with a reference curve generated using homologous blood as previously described [23, 24].

### Cell culture and treatment

#### Rat brain microvascular endothelial cells (BMECs) culture

BMEC isolation was performed as previously described, with minor modifications [25]. Briefly, the meninges-free cortical tissues were dissected from 4-week-old Wistar rats. The cerebral cortex was fully mashed and digested at 37 °C for 40 min with 0.1% type II collagenase (Invitrogen, Camarillo, CA, USA) and 50 U/mL DNaseI (Invitrogen) in Dulbecco’s modified eagle medium (DMEM, Gibco). After the supernatant was removed, the pellet was resuspended and centrifuged at 1000 g for 20 min to isolate the microvessels. The microvessel layer was carefully collected and digested with 0.1% collagenase/dispase and 50 U/mL DNase I in DMEM for 40 min at 37 °C. The pellet was resuspended in PBS, layered over a 33% continuous Percoll gradient and centrifuged at 1000 g for 10 min at 4 °C. The cells were resuspended in complete culture medium and seeded onto collagen I-coated plastic flasks. The culture medium consisted of DMEM supplemented with 20% plasma-derived bovine serum (PDS; First Link, Birmingham, UK), 100 μg/mL heparin, 4 ng/mL basic fibroblast growth factor (bFGF; Invitrogen), 10 μg/mL endothelial cell growth supplement (ECGS; Gibco), 2 mM L-glutamine, and 0.25 μg/mL amphotericin B. The cultures were maintained in a 37 °C incubator with 5% CO_2_, and the culture medium was changed every 2 days. The cells were 70% confluent before experiments were carried out.

#### Primary astrocyte culture

Primary astrocytes were prepared from brain tissues collected from postnatal rats (within 24 h). In brief, after removal of the meninges, the cortex was dissected, cut into 1-mm cubes, and then mechanically dissociated by repeated pipetting. Cells were pelleted and resuspended with glia minimal essential medium (Invitrogen) supplemented with 6 g/L glucose, 1 mM sodium pyruvate, 6% horse serum, and 4% Fetal bovine serum (FBS; Invitrogen). The medium was refreshed every 3–4 days until 100% confluence was observed. All cultures were confluent at the time of the experiments. Using this protocol, greater than 85% of cells were glial fibrillary acidic protein-positive (GFAP+).

#### siRNA-mediated caveolin-1 knockdown

Cultured cells were transfected with either small interfering ribonucleic acid (siRNA) against rat caveolin-1 or with a scrambled control siRNA with no significant homology to any known gene sequences (Ambion, ID#4611) using Lipofectamine™ 2000 according to the manufacturer’s protocol. After 5 h incubation, the medium was replaced with RPMI (Invitrogen) plus 10% FBS, and cells were grown for an additional 48 h before they were used. The efficiency of caveolin-1 silencing was confirmed by isolating RNA (RNeasy Mini kit, Qiagen) from transfected cells and conducting real-time PCR analysis with the Bio-Rad iScript™ One-Step RT-PCR kit with SYBR Green. The cDNA3/GnT-V plasmid was used to transform N152Q mutant which has previously been described elsewhere [26].

### Immunofluorescent staining

The immunofluorescent staining was performed according to previously reported methods [27]. In brief, the rats were anesthetized, first perfused with saline, and then fixed with buffered paraformaldehyde (4%). The brains were removed and immersed in 4% paraformaldehyde overnight for post-fixation and then in 30% sucrose solution overnight. Fifteen-micrometer coronal sections were obtained on a cryostat. The slices were blocked with PBS containing 5% BSA, 10% goat serum, and 0.3% Triton-X 100. Next, the slices were incubated overnight at 4 °C with the primary antibodies: anti-GFAP (1:1000; Millipore, Billerica, MA, USA), anti-NeuN (1:500; Abcam), anti-CD31(1:200; Santa Cruz), anti-CD147(1:500; R&D Systems) or anti-caveolin-1(1:200; BD Biosciences, San Jose, CA, USA), and then Alexa Fluor 488- or 595-labeled secondary antibodies (Molecular Probes Inc., Eugene, OR, USA) for 2 h at room temperature. The tissue sections were washed twice in PBS and then immersed in diphenyl phenylindole (DAPI) solution (1:1000 dilution; Molecular Probes) for 10 min. After being rinsed in distilled water, the sections were fixed with a coverslip using the anti-fade mounting medium.

### Immunoprecipitation and immunoblotting

For immunoprecipitation, HEK293 cells were grown in 60-mm dishes and transiently transfected with 1 μg of plasmids containing HA-caveolin-1 or Flag-CD147 for 48 h in the presence or absence of advanced glycation end products (AGEs, 200 mg/L). Cells were then collected, lysed in immunoprecipitation buffer (1 × PBS, 0.5% Triton X-100, Complete Mini protease inhibitor (Roche Applied Science)) and rotated for 1 h at 4 °C. After centrifugation at 17,500 g for 15 min at 4 °C, the supernatant was collected for immunoprecipitation. Primary monoclonal anti-HA antibody (2 μg, Roche Applied Science) or mouse IgG (2 μg, Santa Cruz) was bound to Dynabeads Protein G (Invitrogen) at room temperature for 30 min by rotation. The bead-antibody complexes were washed in PBS containing 0.01% Tween-20 and then incubated with the above supernatants (500 μL) overnight at 4 °C with rotation. The bead-antibody-antigen complexes were then washed with citrate phosphate buffer (pH 5.0) and boiled in sodium dodecyl sulfate polyacrylamide gel electrophoresis (SDS-PAGE) sample buffer for 5 min to elute the immune complexes. The samples were then separated in gradient gels (5–15%) and immunoblotted with anti-CD147 antibody.

For other immunoblotting experiments, the brain tissues from the striatum supplied by the middle cerebral artery or cells lysed with radioimmunoprecipitation assay buffer (RIPA) containing protease inhibitors (Sigma) were separated by SDS-PAGE and then transferred onto nitrocellulose membranes. The membranes were blocked by 5% (*w*/*v*) skim milk (Becton, Dickinson and Company, Le Pont de Claix, France) for 1 h and then incubated overnight at 4 °C with following primary antibodies: anti-ZO-1 (1:1000; Invitrogen), anti-Occludin (1:1000; Invitrogen), anti-collagen IV (1:2000; Abcam), anti-CD147 (1:1000; R&D Systems), anti-Caroline-1 (1:500; BD Biosciences), and anti-β-actin (1:5000; Sigma). Secondary antibodies conjugated with horseradish peroxidase (HRP) were used, and immunoreactivity was visualized with chemiluminescence (SuperSignal Ultra, Pierce, Rockford, IL, USA). Protein bands were analyzed and quantified using Scion Image system.

### Detection of MMP-2/9 activity

MMP-2/9 activity was detected according to the manufacturer’s instructions (Invitrogen). In brief, equal amounts of protein samples (30 μg) were mixed with SDS sample buffer and loaded onto 10% Tris-glycine gels containing 0.1% gelatin. Human MMP-2/9 standard was also loaded parallelly in each gel. After electrophoresis, gels were washed with renaturing buffer for 1 h and incubated in developing buffer at 37 °C for 48 h. Gels were stained with 0.5% Coomassie blue R-250 for 1 h, and the stain was then removed appropriately. MMP-2/9 band intensity was quantified and normalized to human MMP-2/9 standard.

### Serum CD147 and MMPs detection

Blood was collected 24 h after MCAO by retro-orbital bleeding into polypropylene tubes containing 3.8% trisodium citrate. Platelet-poor plasma was prepared by centrifugation. The amounts of CD147, MMP2, and MMP9 protein in the serum samples were measured using commercial kits (R&D systems, MN, USA) according to the manufacturer’s instructions.

### Statistical analysis

Data is expressed as mean ± SD. Differences between means were analyzed using either one-way or two-way ANOVA followed by Newman-Keuls post hoc testing for pair-wise comparison using SigmaStat v3.5®. Groups of data were considered to be significantly different if *P* < 0.05.

## Results

### Diabetes increases hemorrhagic transformation and BBB disruption after rt-PA treatment in cerebral ischemia

**Fig. 1:**
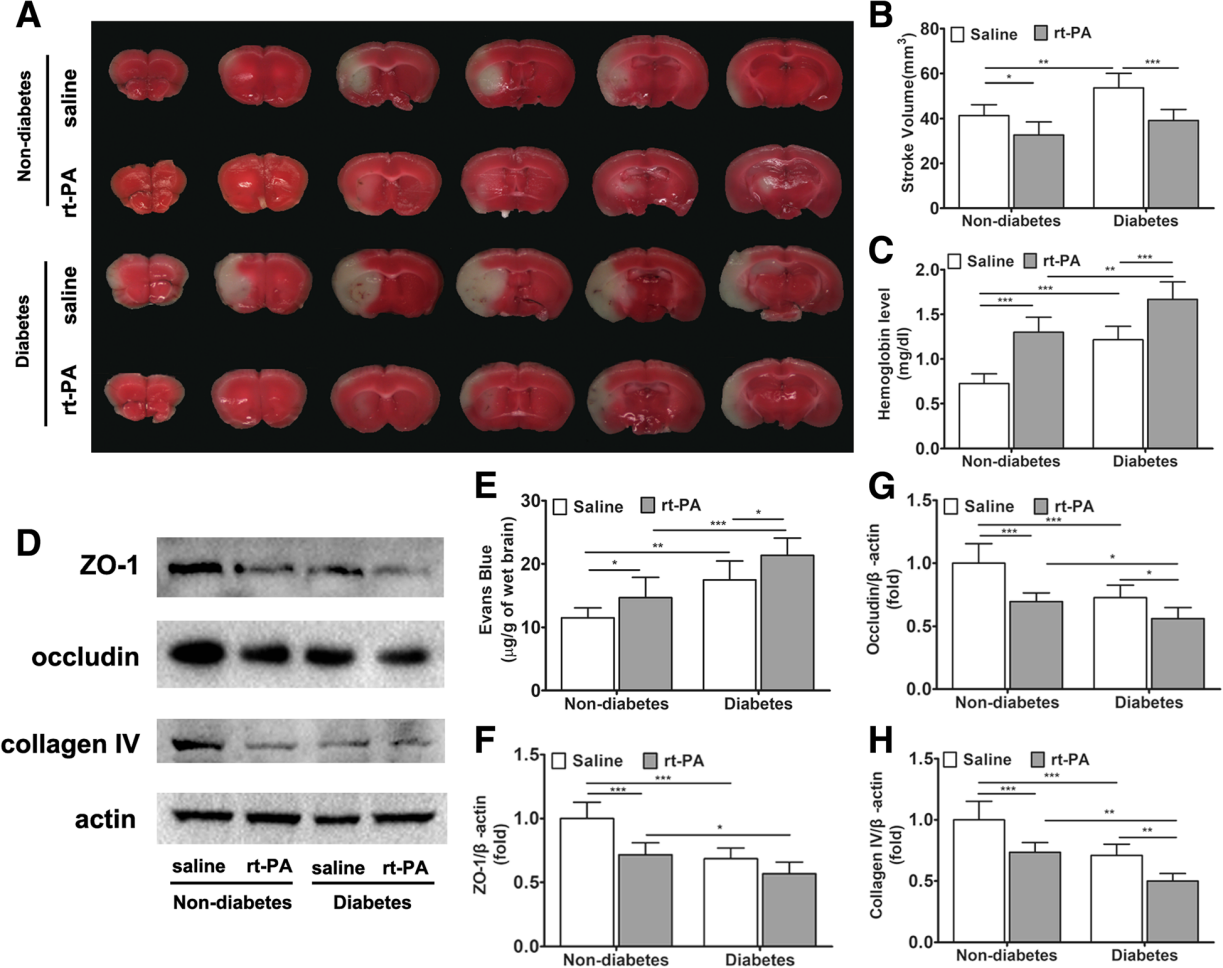
Diabetes increases HT and BBB disruption after rt-PA treatment of cerebral ischemia. Diabetes was induced in rats with streptozotocin. Cerebral Ischemia was induced by middle cerebral artery occlusion and treated with rt-PA or saline as control. Analysis was performed 72 h after ischemic attack. **a** Representative TTC staining images of four groups. **b** Stroke volume evaluated by TTC staining (*n* = 6 in each group). **c** Cerebral hemorrhage evaluated by spectrophotometric hemoglobin assay (*n* = 6 in each group). **d** Representative immunoblots of tight junction proteins zonula occludens-1 (ZO-1) and occludin and collagen IV for each group. **e** BBB permeability assay with Evans blue dye (*n* = 10 in non-diabetic groups, *n* = 6 in diabetic groups). **f**–**h** Protein amounts were quantified from immunoblots and normalized to β-actin amount. Data expressed as mean ± SD and analyzed by two-way ANOVA. One asterisk (*) represents *P* < 0.05, two (**) *P* < 0.01, and three (***) *P* < 0.001. rt-PA recombinant tissue plasminogen activator, BBB blood-brain barrier, HT hemorrhagic transformation, TTC 2,3,5-triphenyltetrazolium chloride, ZO-1 zonula occludens-1

We induced cerebral ischemia in rats by MCAO and treated it with rt-PA. rt-PA thrombolysis was effective in reducing brain infarct volume in both non-diabetic and diabetic rats, but significantly exacerbated intracerebral hemorrhagic transformation (HT) (Fig. 1a–c). Such an undesired side effect became more severe in diabetic rats because the level of HT was already increased by diabetes (Fig. 1c). These results, obtained in rats, are in accordance with our clinical observation in human patients.

Next, we examined the BBB permeability, which is central to HT in ischemic stroke after thrombolytic therapy, using Evans blue dye extravasation assay. The results show that the diabetic rats had higher BBB permeability than non-diabetic controls, while rt-PA therapy increased BBB permeability (Fig. 1e). We further measured the major tight junction proteins, zonula occludens-1 (ZO-1) and occludin, and the major vascular basal membrane protein collagen IV in the peri-infarct region (Fig. 1d, f, g). The results show that in diabetic rats, rt-PA therapy reduced the expression of these proteins in both the non-diabetes and diabetes groups, with the exception of ZO-1. Although these factors were lower in diabetic rats than in the non-diabetic animals. Collectively, we conclude that the exacerbation of HT in rt-PA-treated diabetic rats is due to the increased BBB permeability as a result of accelerated degradation of tight junction proteins and vascular basal membrane proteins.

### Diabetes upregulates CD147 glycosylation and caveolin-1 expression regulating secretion of MMPs

**Fig. 2:**
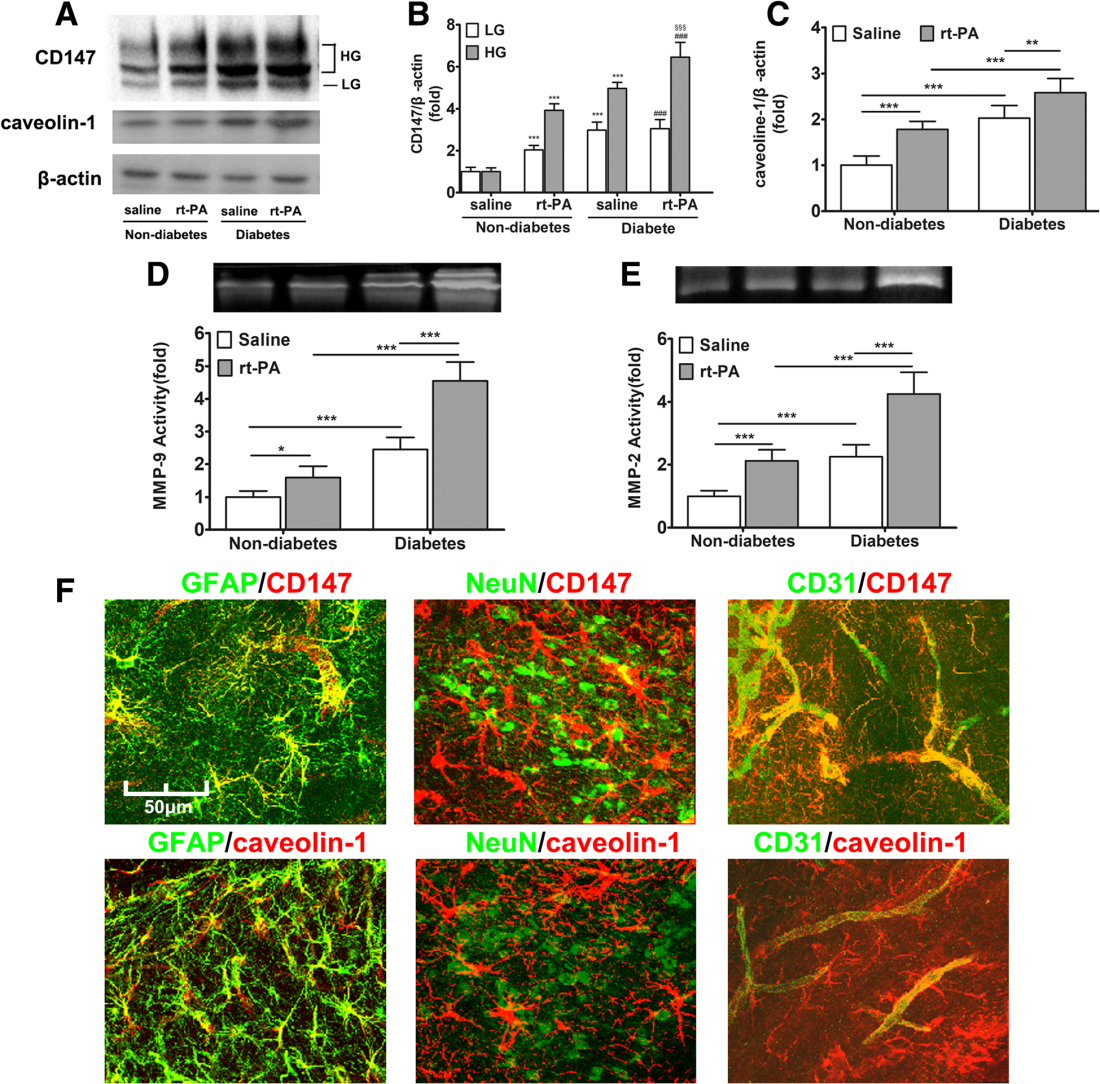
Diabetes promotes CD147 glycosylation and MMPs induction in astrocytes and endothelium. At 72 h after MCAO, brain tissues from ischemic hemispheres were resected and crushed into homogenate. **a** Immunoblot result of ischemic brain tissue homogenate. The protein amounts were densitometrically quantified and normalized to β-actin amount. The expression of both CD147, especially the highly glycosylated form (**b**), and caveolin-1 (**c**) were upregulated by rt-PA and diabetes. The homogenates were also analyzed for MMP-9/2 activities (**d**, **e**). Data expressed as mean ± SEM and analyzed by two-way ANOVA. Asterisk (*) denotes comparison with non-diabetes + saline-treated group, pound (#) with non-diabetes + rt-PA-treated group, and section sign symbol (§) with diabetes + saline-treated group. One asterisk (*) represents *P* < 0.05, two (**) *P* < 0.01, and three (***) *P* < 0.001. **f** Representative immunofluorescence imaging of location of CD147 and caveolin-1 (red color represents for target proteins CD147 or caveolin-1; green color represents for cell markers, GFAP for astrocyte, NeuN for neuron and CD31 for endothelial cell). Abbreviations: rt-PA for recombinant tissue plasminogen activator; MCAO for middle cerebral artery occlusion; MMP for matrix metalloproteinase

It has been reported that rt-PA induces the production of serum MMP-9 and MMP-2, which damage the neurovascular unit and promote BBB disruption [28–30]. We examined the serum MMP-2 and MMP-9 activity at 72 h after MCAO and found that the development of diabetes increased the serum levels of MMP-2 and MMP-9, which were further elevated after rt-PA treatment (Additional file 1: Figure S1A–B). Similarly, CD147, as a key regulator of MMP expression, was upregulated in the serum by diabetes and rt-PA-treatment (Additional file 1: Figure S1C). Although the circulating MMPs contribute to early BBB disruption and HT, the involvement of brain-derived proteases cannot be denied. Therefore, we further examined the MMP-2/9 activity in ischemic brain tissues and CD147 protein in the peri-ischemic region. The results indicated that both the activity of tissue MMPs and CD147 protein were significantly increased by diabetes and rt-PA treatment (Fig. 2a, b, d, e). CD147 occurs in two forms, the 33 kDa LG-CD147 and 40–65 kDa HG-CD147 with different MMP-inducing activity [17]. Both forms were upregulated in diabetic rats, but only HG-CD147 was further upregulated after rt-PA treatment (Fig. 2b). As rt-PA treatment did increase LG-CD147 level in non-diabetic rats, the irresponsiveness of LG-CD147 in diabetic rats seems a result of its complete induction by diabetes. Together, this data implies that diabetes evokes production of brain-derived MMPs by promoting the glycosylation of CD147.

It has been demonstrated that caveolin-1 not only upregulates CD147 glycosylation but also influences the induction of CD147-dependent MMPs in malignant tumor cells [31, 32]. However, whether caveolin-1 is related to diabetes-induced CD147 glycosylation in the central nervous system has not been reported. We examined the level of caveolin-1 expression in the brain and found that this too was also upregulated in association with the expression of HG-CD147 (Fig. 2a, c).

To determine which cells were bearing the increased amount of CD147 and caveolin-1 in the diabetic ischemic brain, we performed immunofluorescent double staining at the peri-ischemic region with cell type-specific markers: GFAP as an astrocyte marker, CD31 as an endothelial cell marker, and NeuN as a neuron marker. As shown in Fig. 2f, in the brain of rt-PA-treated diabetic rats, CD147 co-localized with GFAP-positive astrocytes and CD31-positive endothelial cells, but not with NeuN positive neurons. Consistently, we can determine that caveolin-1 is located in astrocytes and endothelial cells rather than neurons.

### HG-CD147 promotes secretion of MMPs and decreases tight junction protein amounts in brain microvascular endothelial cells (BMECs)

In order to further understand the effects of CD147 glycosylation on the production of MMPs in diabetes, in vitro experiments were conducted. As CD147 is predominantly expressed on endothelial cells and astrocytes, BMECs and primary astrocytes were isolated and cultured. BMECs were exposed to high glucose or AGEs to mimic the diabetic stimulation. The cell layers and conditioned media were then subjected to analysis for CD147 and activity of MMPs, respectively. As shown in Additional file 2: Figure S2, high glucose and AGEs had a similar effect on the glycosylation of CD147, and we subsequently chose AGEs for further investigation.

**Fig. 3:**
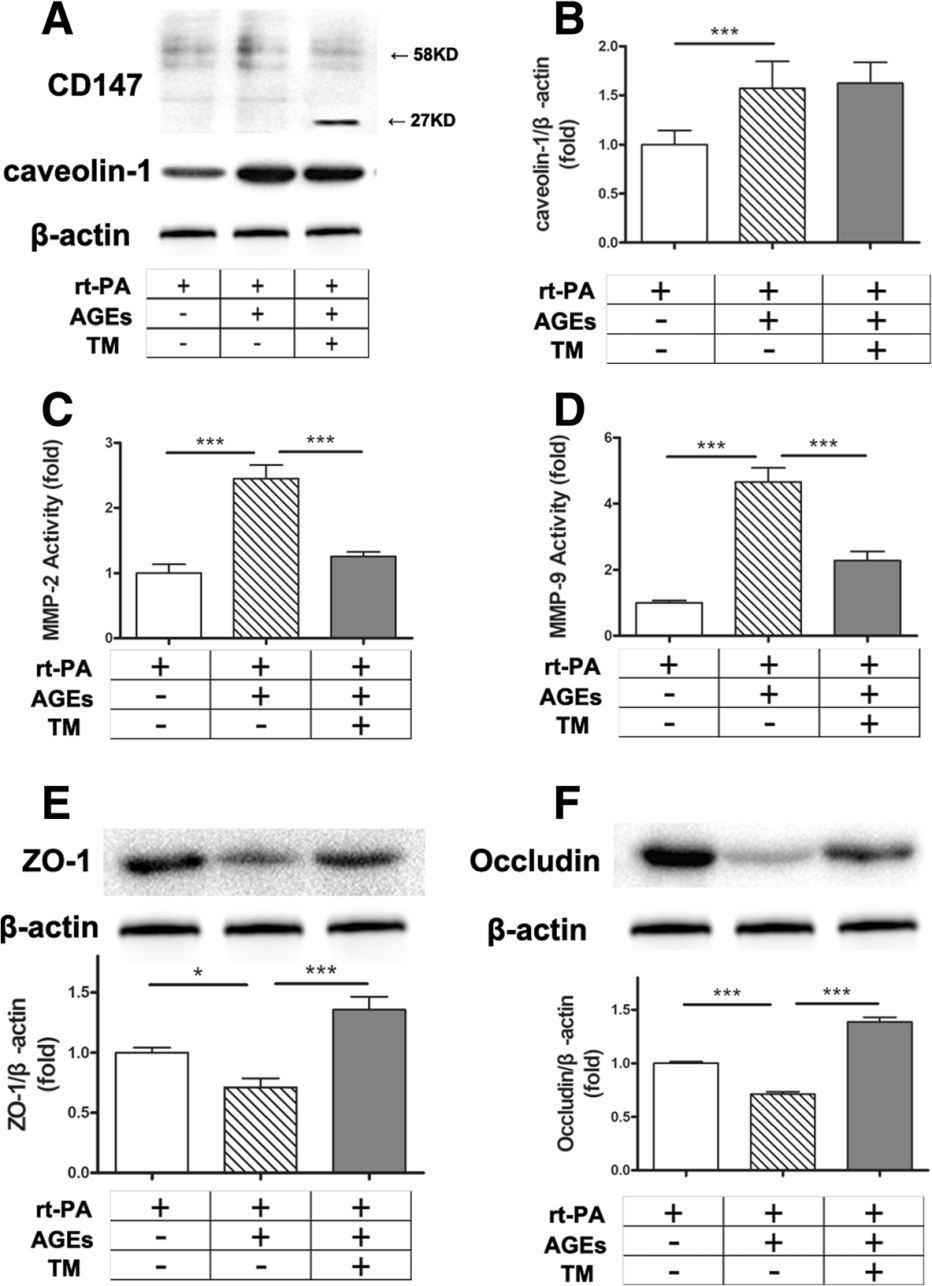
Highly glycosylated CD147 promotes secretion of MMPs and decreases tight junction protein amount in BMECs. **a** BMECs were cultured and stimulated with rt-PA (10 μg/mL) in the presence or absence of AGEs (200 μg/mL for 48 h) and the N-glycosylation inhibitor tunicamycin (TM, 15 μg/mL for 48 h, Calbiochem, San Diego, CA). The cell layers were then subjected to immunoblot assay for CD147 and caveolin-1. A representative blot result is shown. **b** Densitometric quantification results of caveolin-1. **c**, **d** MMPs activities in the conditioned media. **e**, **f** Representative immunoblot and densitometric quantification results of occludin and ZO-1. Data expressed as mean ± SEM and analyzed by one-way ANOVA test. One asterisk (*) represents *P* < 0.05, two (**) *P* < 0.01, and three (***) *P* < 0.001. rt-PA for recombinant tissue plasminogen activator; AGEs for glycation end products; MMP for matrix metalloproteinase; ZO-1 for zonula occludens-1

AGEs-rt-PA treatment significantly increased the expression of CD147 proteins in cultured BMECs, of which most were HG-CD147 (40–65 kDa; Fig. 3a). In addition, AGEs-rt-PA treatment increased the secretion of MMP-2 and MMP-9 by 2.5 and fivefold, respectively (Fig. 3c, d). Furthermore, the amounts of two major tight junction proteins, ZO-1, and occludin in BMECs decreased significantly after AGEs-rt-PA treatment (Fig. 3e, f). These results are consistent with those observed in rat models, suggesting that the BMEC-derived CD147 and MMPs (MMP-9 in particular) may participate in the BBB disruption as a consequence of ischemic stroke and rt-PA-induced hemorrhagic complications.

To confirm the role of CD147 glycosylation in MMP induction, we first suppressed the N-glycosylation in BMECs using the general inhibitor tunicamycin (TM). TM treatment was found to successfully deplete N-glycan from CD147, since most CD147 occurred as a 27 kDa band on immunoblot, representing the non-glycosylated form of CD147 (Fig. 3a). As expected, TM treatment significantly suppressed the induction of the activities of MMPs and prevented the degradation of tight junction proteins in BMECs (Fig. 3c–f).

**Fig. 4:**
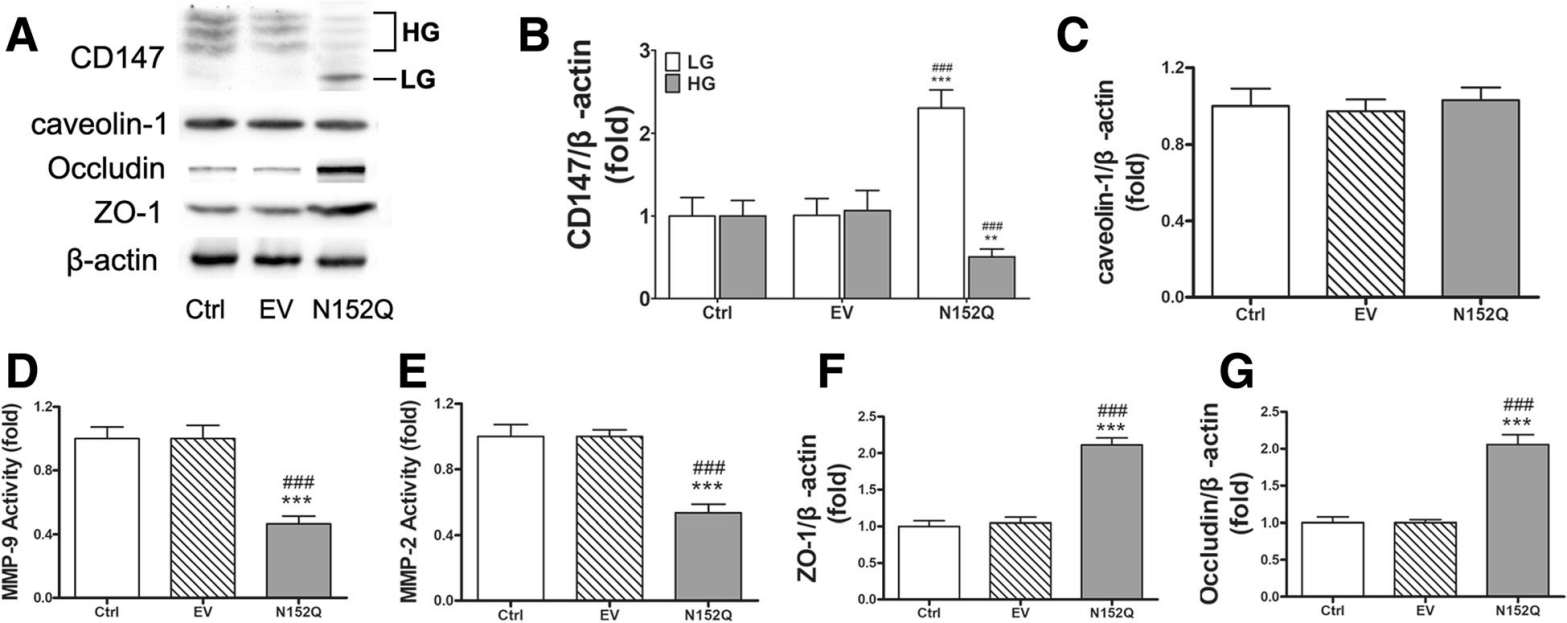
Overexpression of N-glycosylation defective CD147 mutant N152Q blocked MMPs production in BMECs. **a** Representative Western blots of CD147, caveolin-1, occludin, and ZO-1. **b**, **c** Densitometric quantification results of CD147 and caveolin-1 in control and empty vector- or N152Q mutant-transfected BMECs. **d**, **e** MMP-2/9 activity in the conditioned media. **f**, **g** Densitometric quantification results of occludin and ZO-1. Data expressed as mean ± SEM and analyzed by one-way ANOVA. Asterisk (*) denotes comparison with the control group and pound (#) with the empty vector group. One asterisk (*) represents *P* < 0.05, two (**) *P* < 0.01, and three (***) *P* < 0.001. EV empty vector, BMEC brain microvascular endothelial cell, MMP matrix metalloproteinase, ZO-1 zonula occludens-1

As tunicamycin treatment lacks target protein specificity, we further utilized the specific dominant negative form of CD147, the N152Q mutant. The overexpression of N152Q in BMECs potently reduced the portion of HG-CD147 (Fig. 4a, b) and was found to be as effective as TM treatment in suppressing MMPs and protecting tight junction proteins (Fig. 4d–g).

Taken together, these results demonstrated clearly that HG-CD147 is a key molecule in the diabetes-related BBB disruption and thrombolytic therapy-induced hemorrhagic transformation.

### AGEs with rt-PA increases CD147 glycosylation and MMP-2 activity in astrocytes

**Fig. 5:**
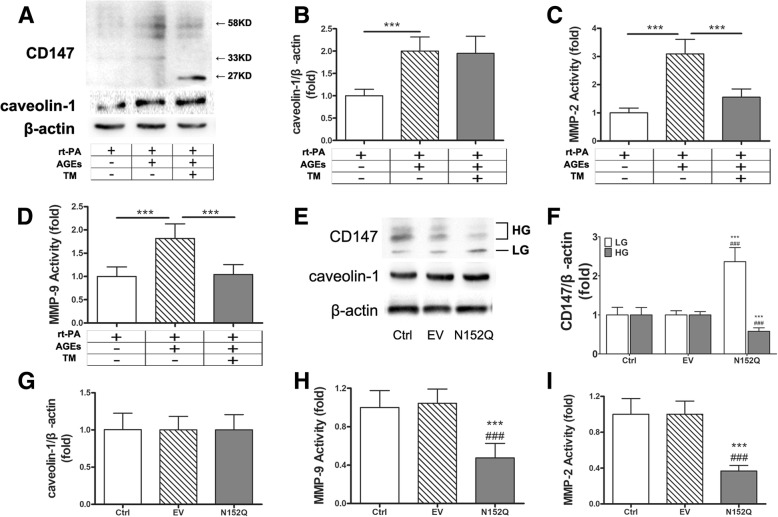
Highly glycosylated CD147 promotes MMPs activity in primary astrocytes. **a** Representative immunoblots of CD147 and caveolin-1 in AGEs and tunicamycin-treated primary astrocytes. **b** Densitometric quantification results of caveolin-1. **c**, **d** MMP-2/9 activity in the conditioned media. One asterisk (*) represents *P* < 0.05, two (**) *P* < 0.01, and three (***) *P* < 0.001. **e** Representative Western blots of CD147 and caveolin-1 in empty vector- or CD147 N152Q mutant-transfected astrocytes. **f**, **g** Densitometric quantification results of CD147 and caveolin-1. **h**, **i** MMP-2/9 activity in the conditioned media. Data expressed as mean ± SEM and analyzed by one-way ANOVA. Asterisk (*) denotes comparison with the control group and pound (#) with the empty vector group. rt-PA recombinant tissue plasminogen activator, AGEs glycation end products, TM tunicamycin, EV empty vector, Ctrl control, MMP matrix metalloproteinase, ZO-1 zonula occludens-1

Based on immunofluorescence staining results, CD147 and caveolin-1 are mainly located on endothelial cells and astrocytes. To know whether CD147 glycosylation could also influence the production of MMPs in astrocytes, we isolated primary astrocytes and treated them with rt-PA in the presence or absence of AGEs. AGEs-rt-PA treatment significantly increased the glycosylation of CD147 and increased the activity of MMPs (Fig. 5a, c, d). Unlike in BMECs, more MMP-2 was induced than MMP-9 in astrocytes (Fig. 5c, d—a three- to fourfold increase vs 1.8-fold increase).

Tunicamycin treatment and CD147 N152Q transfection were also applied to astrocytes. The results showed that either pharmacological blockage or genetic interference of the glycosylation of CD147 significantly inhibited the induction of MMPs (Fig. 5c, d, h, i). This data was consistent with those in BMECs.

### Caveolin-1 binds to CD147 and upregulates its glycosylation

**Fig. 6:**
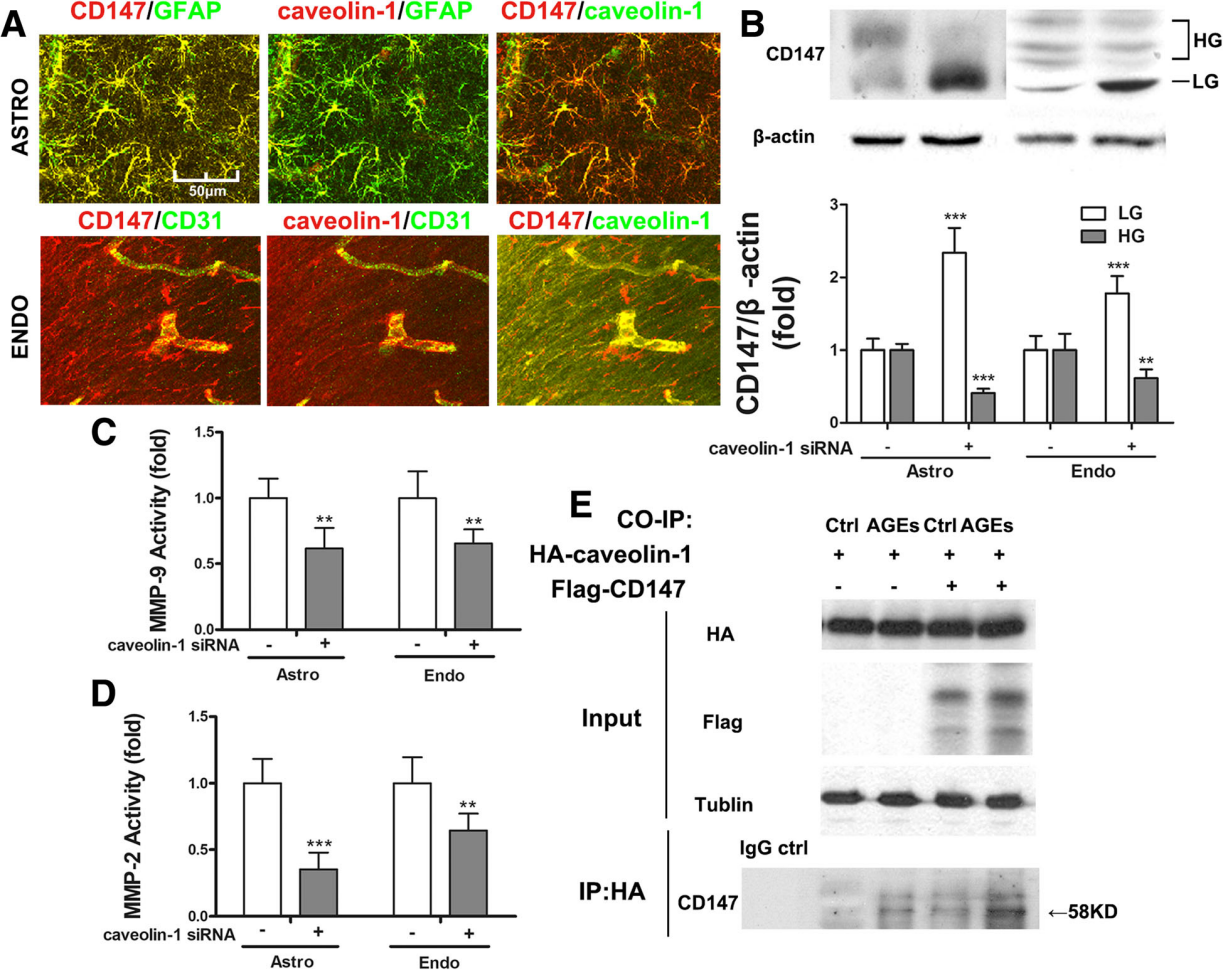
Caveolin-1 binds to CD147 and upregulates its glycosylation. **a** CD147 and caveolin-1 co-localize in astrocytes and endothelium. **b** Caveolin-1 silencing by siRNA downregulates HG-CD147 in astrocytes and endothelial cells treated with AGEs and rt-PA. **c**, **d** Decrease of HG-CD147 reduces MMPs activities. Data expressed as mean ± SD and analyzed by one-way ANOVA test. One asterisk (*) represents *P* < 0.05, two (**) *P* < 0.01, and three (***) *P* < 0.001. **e** HEK293 cells were transfected with HA-caveolin-1 and FLAG-CD147 constructs. The whole cell lysates were subjected to immunoblot analysis (input). In addition, the whole cell lysates were first immunoprecipitated with anti-HA antibody and then detected with anti-CD147 antibody (IP:HA). ASTRO astrocyte, ENDO endothelial cell; HG highly glycosylated, LG lowly glycosylated, MMP matrix metalloproteinase, CO-IP co-immunoprecipitation

Caveolin-1 is a scaffold protein capable of collecting signaling molecules, including CD147, into the caveolae and regulating their activities. It has been reported that caveolin-1 may participate in the process of CD147 glycosylation [31, 33]. However, whether caveolin-1 affects CD147 glycosylation in DM is yet to be verified. In this study, we found that upregulated caveolin-1 and CD147 were co-localized on both astrocytes and endothelium in the diabetic model (Fig. 6a), indicating a potential interaction between these two proteins. In addition, the specific suppression of N-glycosylation of CD147 did not impact the expression of caveolin-1 (Figs. 4c and 5g), implying that caveolin-1 functions as an upstream of CD147. To corroborate this, we knocked down caveolin-1 expression (by 80%, data not shown) using siRNA in both astrocytes and BMECs treated with AGEs and rt-PA. We found that a significant proportion of highly glycosylated CD147 was changed into the lowly glycosylated form (Fig. 6b). Furthermore, the activity of MMPs decreased significantly (Fig. 6c, d). These results indicate that caveolin-1 is required for the glycosylation of CD147 in astrocytes and endothelium under rt-PA treatment and diabetic circumstance.

We then examined whether caveolin-1 directly interacts with CD147. To this end, we transfected HEK 293 cells (due to their high transfection efficiency) with Flag-CD147 and HA-caveolin-1 constructs in the presence or absence of AGEs. The expression of these fusion proteins was confirmed by immunoblotting with whole cell lysates (Fig. 6e, input panel). The cell lysates were then immunoprecipitated with anti-HA antibody or matched IgG followed by immunoblotting with CD147 antibody. The results indicated that CD147 was pulled down by anti-HA antibody but not matched IgG (Fig. 6e, ip panel). More CD147 protein was pulled down in the presence of AGEs, which appears to be a result of the increased amount of HG-CD147 after AGEs stimulation (Fig. 6e, ip panel). We conclude that caveolin-1 binds directly to CD147 and regulates its glycosylation.

### Exendin-4 ameliorates rt-PA-induced HT by downregulating the HG-CD147/MMPs pathway

**Fig. 7:**
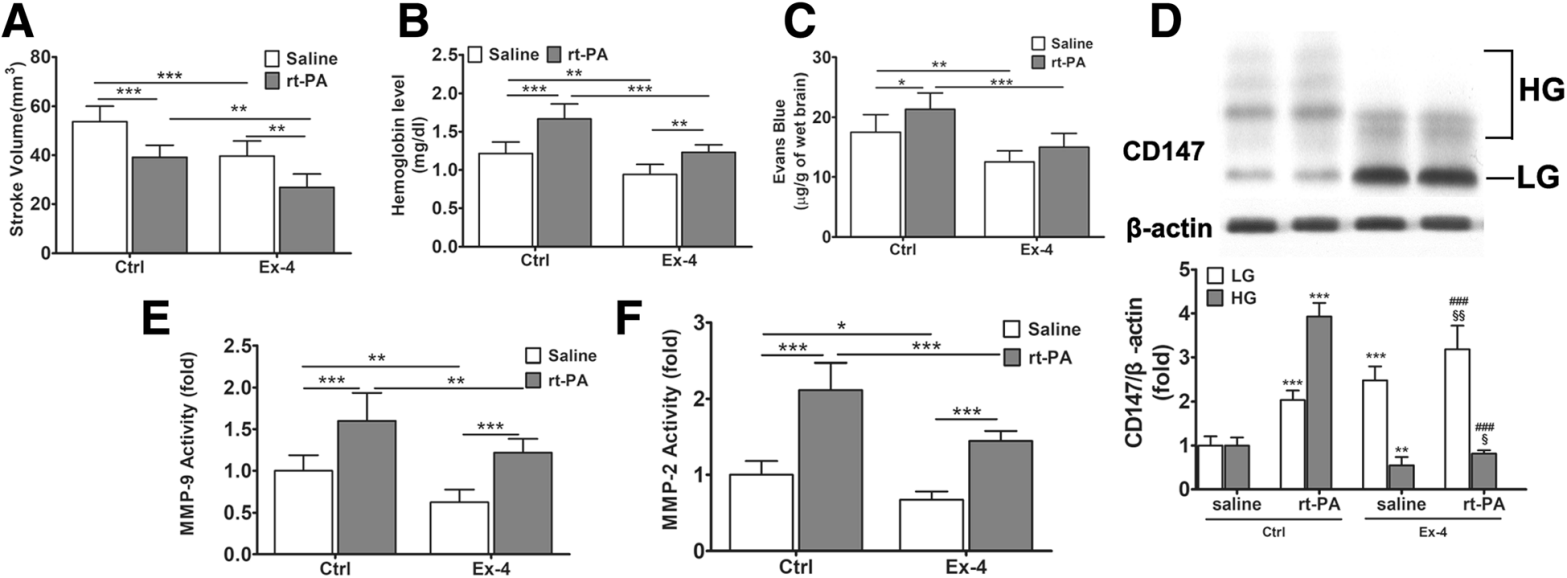
Ex-4 ameliorates rt-PA-induced hemorrhagic transformation by downregulating HG-CD147/MMPs pathway. Ex-4 is a GLP-1R agonist that promotes insulin secretion. **a**, **b** Ex-4 treatment reduces the stroke volume and HT in both saline and rt-PA groups. **c** Ex-4 shows a protecting effect on BBB integrity against rt-PA in Evans blue dye assay. Data expressed as mean ± SD. **d** Representative immunoblots and densitometric quantifications of CD147. Asterisk denotes comparison with control group without rt-PA treatment, number sign denotes comparison with control group with rt-PA treatment, and section sign denotes comparison with Ex-4-treated group without rt-PA treatment. **e**, **f** MMP-2/9 activity. Data expressed as mean ± SEM and analyzed by two-way ANOVA. One asterisk (*) represents *P* < 0.05, two (**) *P* < 0.01, and three (***) *P* < 0.001. rt-PA recombinant tissue plasminogen activator, Ex-4 Exendin-4, MMP matrix metalloproteinase, HG highly glycosylated, LG lowly glycosylated, GLP-1R glucagon-like peptide-1 receptor

Our data indicates that diabetes promotes secretion of MMPs through CD147 glycosylation, causing BBB damage. Upon rt-PA treatment, the damage is further exacerbated and leads to significant HT. Considering the possible clinical application of our findings, we examined whether diabetes medication could protect BBB and ameliorate thrombolysis-induced HT without increasing the risk of hypoglycemia. Exendin-4 (Ex-4) is an endogenous glucagon-like peptide-1 receptor (GLP-1R) agonist that promotes insulin secretion. Our previous research shows that Ex-4 decreases warfarin-related HT in diabetic rats by inhibiting MMP-9 activity [21]. In this study, we examined the effect of Ex-4 on rt-PA-related HT in diabetic rats and found that it was also effective in reducing stroke volume (Fig. 7a) and alleviating HT (Fig. 7b). The permeability of BBB elevated by rt-PA treatment was reversed by Ex-4 (Fig. 7c), which may be attributed to the lowered activity of MMPs (Fig. 7e, f) and protection of tight junctions (data not shown). Finally, we examined the CD147 concentration in the brain tissue and found Ex-4 reduced the HG/GL-CD147 ratio in both groups with or without rt-PA (Fig. 7d), supporting the notion that Ex-4 inhibits MMP-2/9 possibly through regulating the HG/GL-CD147 ratio.

## Discussion

**Fig. 8:**
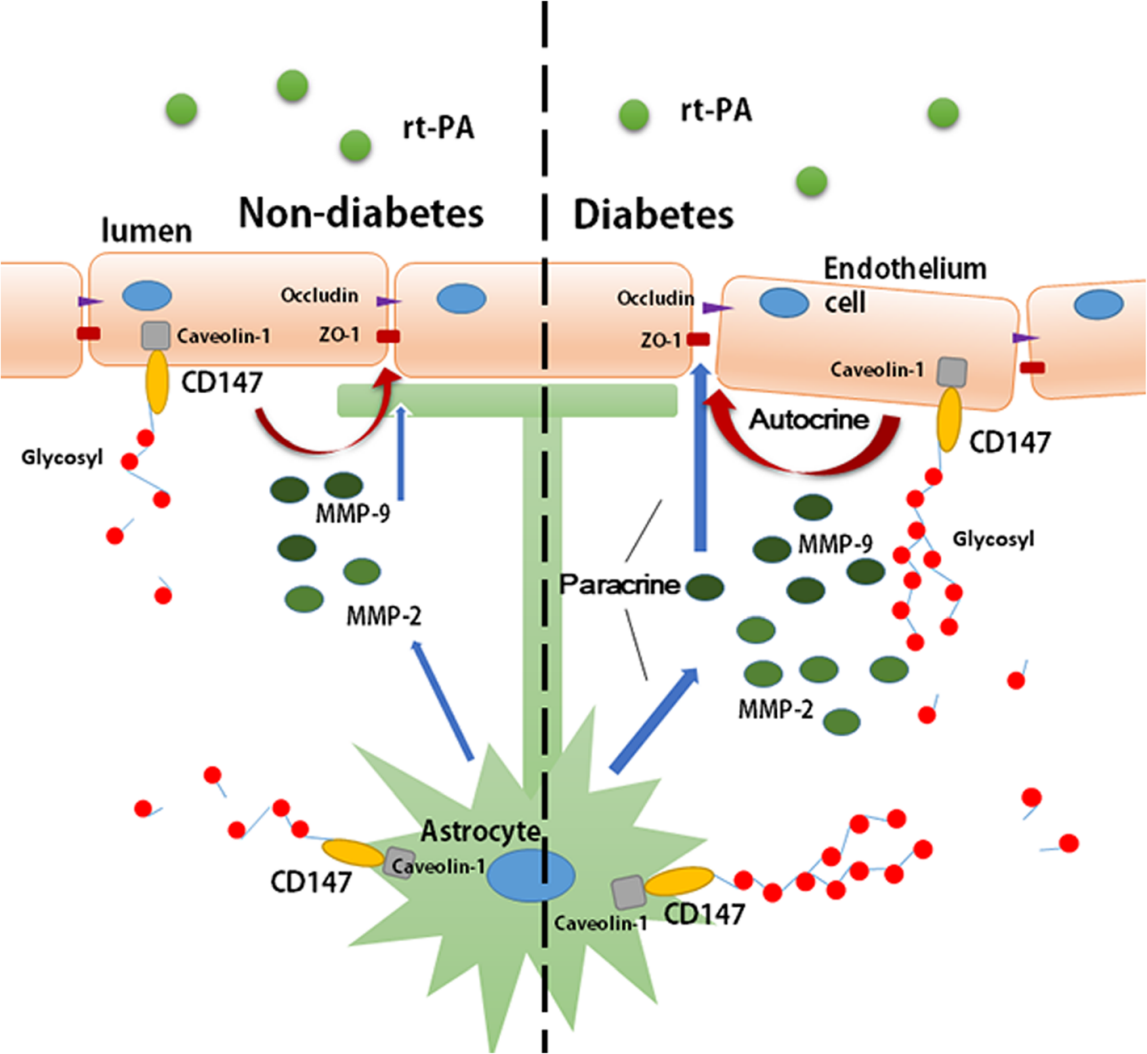
Abridged general view. Diabetes upregulated the expression of caveolin-1 and the glycosylation of CD147 in both astrocytes and endothelium and further elevated the activities of MMPs, especially MMP-2/9. Higher MMP activity accelerates the degradation of BBB components and ultimately leads to erythrocyte leakage after rt-PA treatment of acute cerebral ischemia

Diabetes not only increases the risk of onset of cerebral vascular disease, but also elevates the risk of a poorer prognosis for those who receive thrombolysis therapy. Moreover, diabetes limits the time window for application of thrombolysis therapy. Acute ischemic stroke patients with a history of both diabetes and stroke can only receive thrombolytic therapy in the 3 h from onset due to their higher risk of symptomatic intracranial hemorrhage, while the therapeutic time window expands to 4.5 h to those without diabetes [34]. Our research aims to clarify how diabetes increases HT after thrombolytic therapy and to search for possible effective treatments. Our results indicated that DM promoted the glycosylation of CD147 and the expression of caveolin-1, increased the induction of MMPs, aggravated the disruption of BBB, and eventually exacerbated hemorrhagic transformation of acute ischemic stroke after rt-PA treatment (Fig. 8). Decreasing the CD147 glycosylation could be a potential way to alleviate DM-associated HT after thrombolysis therapy.

Numerous researchers have suggested that MMPs play a critical role during vascular remodeling in diabetes, which is closely related to the comorbidities of cardiovascular diseases. Meanwhile, the study of the mechanism on how DM upregulates MMPs is scarce. Our work indicates that CD147 is essential for the activity of MMPs in DM. Rats with DM showed upregulated expression of CD147, especially HG-CD147 on astrocytes and endothelium, followed by elevated MMP activity in both ischemic brain tissue and serum. CD147, as an important MMP-regulating protein, has been extensively studied in tumor research and has been proven to participate in multiple processes, including cell proliferation and tumor metastasis. In CNS biology, alongside induction of MMPs, CD147 is also involved in homeostasis [35]. But the function of CD147 in ischemic stroke under the diabetic condition is poorly understood. Portik-Dobos et al. explored CD147 function in cardiac artery specimens from DM patients and concluded that CD147 decreased the activity of MMPs, which might contribute to the increased collagen deposition and pathological remodeling observed in diabetes [36]. In contrast, Abu et al. suggested that CD147 upregulated the activity of MMPs and that the CD147/MMPs/VEGF pathway was involved in proliferative diabetic retinopathy angiogenesis [37]. Our conclusive results are in accordance with the latter study, and the reason may be that CD147 function differs between tissues.

In the pathological progression of stroke, gelatinases including MMP-2 and MMP-9, possess the ability to activate numerous pro-inflammatory agents, including chemokine CXCL-8, interleukin 1β, and tumor necrosis factor α [38, 39]. Moreover, they facilitate the movement of leukocytes across the endothelium by digesting collagen VI and tight junction proteins [40]. We observed that AGEs stimulation induced more MMP-9 in BMECs, while inducing more MMP-2 in primary astrocytes, indicating that MMP-9 was predominantly secreted by endothelial cells and MMP-2 was secreted mainly by astrocytes. As MMP-9 is the major subversive of the BBB, endothelial cells are considered to be the main target for the protection of the neurovascular unit following stroke.

The glycosylation of CD147 is essential to its physiological and pathological functions. Multiple pieces of evidence show that CD147 exists in two forms, HG-CD147 (~ 40–65 kDa) and LG-CD147 (~ 33 kDa), with the former thought to be the functioning form for inducing MMPs [17]. The main post-translational modification of CD147 is through N-glycosylation. CD147 contains three Asn glycosylation sites in the extracellular domain which contribute equally to CD147 glycosylation [32]. Besides tunicamycin, we also used the N152Q mutant to suppress CD147 glycosylation. The N152Q mutant has been reported to cause improper intracellular accumulation of CD147 and impair its MMP-inducing activity [25]. Previous studies about caveolin-1-CD147 interaction have proven controversial. Jia et al. claimed that caveolin-1 expression increased the HG/LG-CD147 ratio [41], while Tang et al. drew the opposite conclusion [32]. Our results show that the expression of caveolin-1 and HG-CD147 was upregulated in DM rats with ischemic stroke. Suppression of CD147 glycosylation did not affect the expression of caveolin-1, while suppression of caveolin-1 expression reduced the HG/LG-CD147 ratio. Co-immunoprecipitation results further confirmed the direct interaction between these two proteins. These observations substantially indicate that caveolin-1 is an upstream of CD147 and upregulates the HG/LG-CD147 ratio in brain tissue of DM rats after stroke.

In vitro experiments suggested that a low HG/LG-CD147 ratio inhibits the activity of MMPs and prevents tight junction proteins from degradation, thus protecting the integrity of the BBB. We further verified that Ex-4, a GLP-1R agonist, decreases stroke volume and HT by reversing the HG/LG-CD147 ratio and suppressing the activity of MMPs in vivo. A previous study suggested that Ex-4 protects against post-myocardial infarction remodeling via specific actions on inflammation and the extracellular matrix [42]. Our previous work also showed that Ex-4 can reduce warfarin-associated HT in mice through PI3K/Akt/GSK-3β pathway [21]. In this work, we further provide evidence that Ex-4 protects BBB integrity through reducing the secretion of MMPs. More importantly, we demonstrated that Ex-4 inhibits the process of CD147 glycosylation. GLP-1R agonist is a novel diabetic medication, which has been proven to be safer than basic insulin with a lower risk of hypoglycemia [43]. Although Fan et al. demonstrated that insulin combined with rt-PA thrombolysis could significantly reduce HT [44], its clinical application is limited by its lack of therapeutic safety. Thus, we believe that GLR-1R agonist may offer a potential strategy for the treatment of thrombolytic patients with diabetes or stress hyperglycemia, which deserves further verification in clinical trials.

## Conclusion

The glycosylation of CD147 is essential in DM-associated HT after thrombolysis therapy. Our research gives a clue that decreasing the HG/LG-CD147 ratio could be a way to protect the integrity of the neurovascular unit and promote better outcome following ischemic stroke treated with rt-PA.

## Additional files

Additional file 1: Figure S1. Diabetes upregulates serum levels of MMPs in rats. (A) Concentration of serum MMP-9 protein. (B) Concentration of serum MMP-2 protein. (C) Concentration of serum CD147 protein. Data expressed as mean ± SD and analyzed by two-way ANOVA. *P < 0.05 compared with non-diabetes saline-treated group, ^#^*P* < 0.05 compared with non-diabetes rt-PA-treated group. (TIF 1557 kb)

Additional file 2: Figure S2. High glucose and AGEs equally upregulated the expression of CD147. Brain microvascular endothelial cells (BMECs) were isolated and cultured, followed by immunoblot analysis for CD147 and actin as internal control. The densitometric quantification results are presented. Data expressed as mean ± SD and analyzed by two-way ANOVA. *P < 0.05 compared with non-diabetes saline-treated group, ^#^*P* < 0.05 compared with non-diabetes rt-PA-treated group. (TIF 122 kb)

## Abbreviations

AGEs: Advanced glycation end products
BBB: Blood-brain barrier
bFGF: Basic fibroblast growth factor
BMEC: Brain microvascular endothelial cell
CBF: Cerebral blood flow
DM: Diabetes mellitus
DMEM: Dulbecco’s modified eagle medium
ECGS: Endothelial cell growth supplement
Ex-4: Exendin-4
GFAP: Glial fibrillary acidic protein
GLP-1R: Glucagon-like peptide-1 receptor
HG-: Highly glycosylated
HRP: Horseradish peroxidase
HT: Hemorrhagic transformation
IgSF: Immunoglobulin superfamily
LG-: Lowly glycosylated
MCAO: Middle cerebral artery occlusion
MMP: Matrix metalloproteinase
OD: Optical density
PDS: Plasma-derived bovine serum
RIPA: Radioimmunoprecipitation assay buffer
rt-PA: Recombinant tissue plasminogen activator
TM: Tunicamycin
TTC: 2,3,5-Triphenyltetrazolium chloride
ZO-1: Zonula occludens-1
