# Supplementary figures and images for: Highly glycosylated CD147 promotes hemorrhagic transformation after rt-PA treatment in diabetes: a novel therapeutic target?
Source: J Neuroinflammation. 2019 Apr 5;16:72. doi: 10.1186/s12974-019-1460-1 (PMC6449915; doi:10.1186/s12974-019-1460-1)

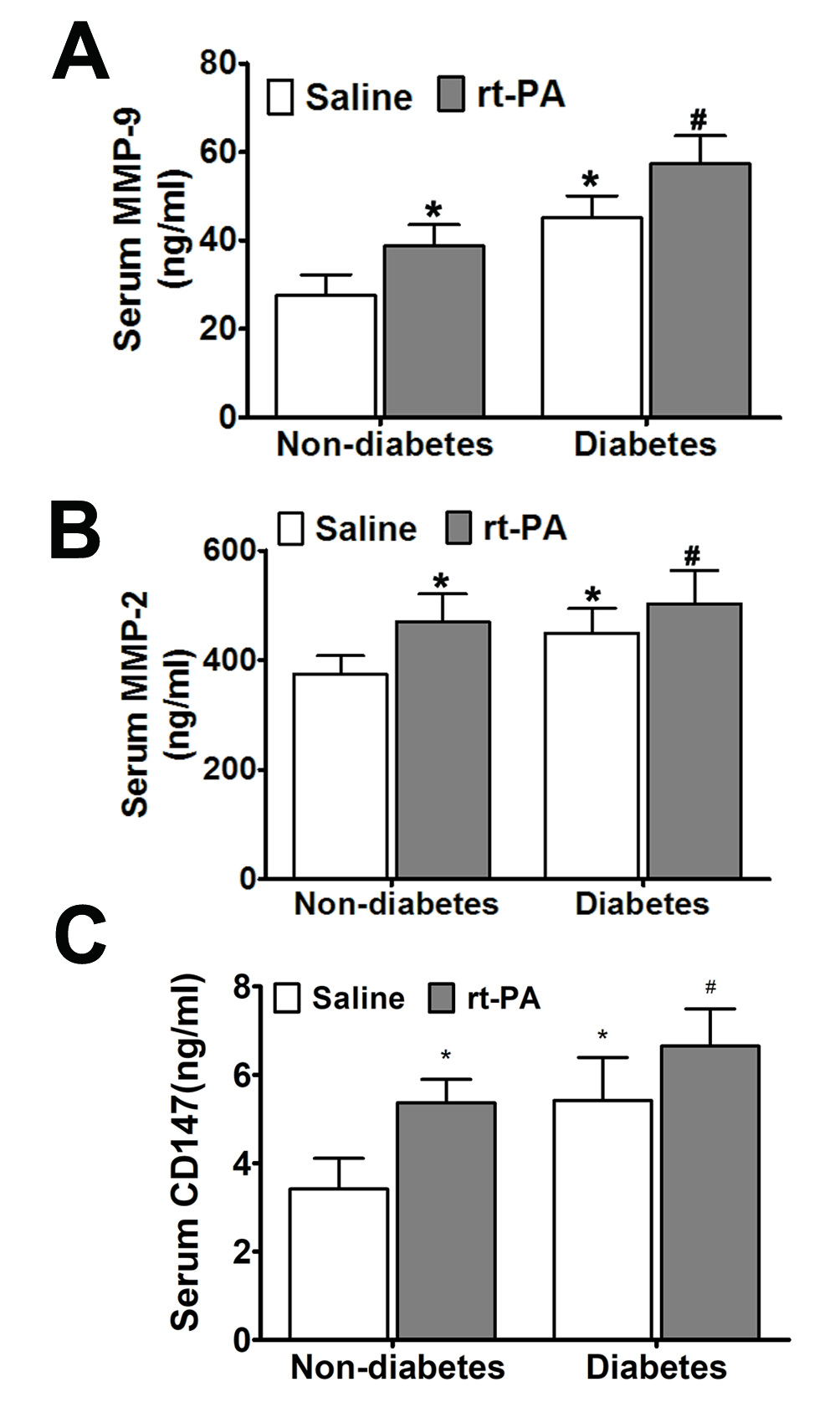

Supplement: Supplementary file 1 — Figure S1. Diabetes upregulates serum levels of MMPs in rats. (A) Concentration of serum MMP-9 protein. (B) Concentration of serum MMP-2 protein. (C) Concentration of serum CD147 protein. Data expressed as mean ± SD and analyzed by two-way ANOVA. *P < 0.05 compared with non-diabetes saline-treated group, #P < 0.05 compared with non-diabetes rt-PA-treated group. (TIF 1557 kb) [file 12974_2019_1460_MOESM1_ESM.tif]

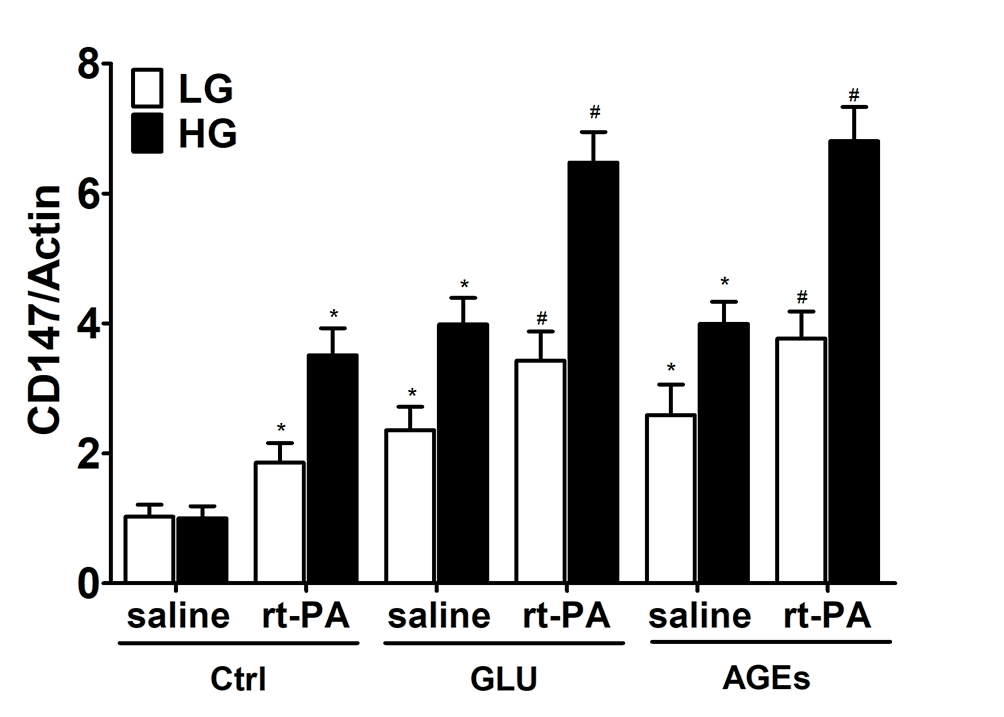

Supplement: Supplementary file 2 — Figure S2. High glucose and AGEs equally upregulated the expression of CD147. Brain microvascular endothelial cells (BMECs) were isolated and cultured, followed by immunoblot analysis for CD147 and actin as internal control. The densitometric quantification results are presented. Data expressed as mean ± SD and analyzed by two-way ANOVA. *P < 0.05 compared with non-diabetes saline-treated group, #P < 0.05 compared with non-diabetes rt-PA-treated group. (TIF 122 kb) [file 12974_2019_1460_MOESM2_ESM.tif]
